# The association between SGLT2 inhibitors and new-onset arrhythmias: a nationwide population-based longitudinal cohort study

**DOI:** 10.1186/s12933-020-01048-x

**Published:** 2020-06-05

**Authors:** Hung-Yi Chen, Jing-Yang Huang, Wun-Zhih Siao, Gwo-Ping Jong

**Affiliations:** 1 Department of Pharmacy, China Medical University and China Medical University Beigang Hospital, Yunlin County, Taiwan, ROC; 2 Department of Medical Research, Chung Shan Medical University Hospital, Taichung, Taiwan, ROC; 3 Division of Cardiology, Department of Internal Medicine, Chung Shan Medical University Hospital and Chung Shan Medical University, Taichung, Taiwan, ROC

**Keywords:** Sodium–glucose co-transporter inhibitors, Diabetes mellitus, Arrhythmias

## Abstract

**Background:**

Clinical trials have shown the cardiovascular protective effect of sodium–glucose cotransporter-2 (SGLT2) inhibitors and reduced hospitalization for heart failure. However, no study has investigated the association between SGLT2 inhibitors and the risk of arrhythmias. This study aimed to evaluate the risk of new-onset arrhythmias (NOA) and all-cause mortality with the use of SGLT2 inhibitors.

**Methods:**

This was a population-based cohort study utilizing Taiwan’s National Health Insurance Research Database. Each patient aged 20 years and older who took SGLT2 inhibitors was assigned to the SGLT2 inhibitor group, whereas sex-, age-, diabetes mellitus duration-, drug index date-, and propensity score-matched randomly selected patients without SGLT2 inhibitors were assigned to the non-SGLT2 inhibitor group. The study outcome was all-cause mortality and NOA.

**Results:**

A total of 399,810 patients newly diagnosed with type 2 DM were enrolled. A 1:1 matching propensity method was used to match 79,150 patients to 79,150 controls in the non-SGLT2 inhibitors group for analysis. The SGLT2 inhibitor group was associated with a lower risk of all-cause mortality [adjusted hazard ratio (aHR) 0.547; 95% confidence interval (CI) 0.482–0.621; *P* = 0.0001] and NOA (aHR 0.830; 95% CI 0.751–0.916; *P* = 0.0002).

**Conclusions:**

Patients with type 2 DM prescribed with SGLT2 inhibitors were associated with a lower risk of all-cause mortality and NOA compared with those not taking SGLT2 inhibitors in real-world practice.

## Background

The global incidence and prevalence of diabetes mellitus (DM) increased over the past two decades and caused much health burden worldwide [1, 2]. DM is associated with an increased risk of atherosclerotic cardiovascular diseases such as coronary artery disease, myocardial infarction, and peripheral artery occlusive disease. Otherwise, patients with DM are prone to develop arrhythmias, which contributed to autonomic, electrical, and structural remodeling, as well as insulin resistance and glycemic fluctuations [3, 4]. DM is also a strong risk factor for sudden cardiac death and responsible for arrhythmic deaths [5, 6]. Once a diabetic population develops arrhythmias, they have a worse prognosis [7]. Sodium–glucose cotransporter-2 (SGLT2) inhibitors as antihyperglycemic drugs are proven to have a cardiovascular protective effect in reducing cardiovascular death and hospitalization for heart failure (hHF) in large randomized trials [8–10]. Animal studies and clinical trials have shown a sympathoinhibitory effect by SGLT2 inhibitors, which play an important role in arrhythmogenesis [11–14]. SGLT2 inhibitors also reduce cardiac fibrosis and left ventricular hypertrophy, which serves as a substrate for arrhythmia development [15, 16]. However, no study has investigated the association of risk of arrhythmias and the use of SGLT2 inhibitors in diabetes populations until now in real-world practice. The purpose of the present study was to evaluate the risk of arrhythmias and all-cause mortality associated with prescription of SGLT2 inhibitors in a nationwide cohort study of diabetic patients in Taiwan.

## Methods

### Data source

This study was approved by the Institutional Review Board of Chung Shan Medical University Hospital, Taiwan. This study extracted data from the National Health Insurance (NHI) program, which is a compulsory universal health insurance program in Taiwan. The NHI database provides comprehensive medical care coverage to more than 99% of Taiwanese residents. It stores information including claim forms and contains patient identification such as numbers, sex, age, three diagnostic codes, medical expenditures, hospital, physician information, and prescriptions such as the drug quantity and expenditure, drug dose, operations, and treatments. Informed consent was waived owing to the retrospective nature of the study; each patient in the NHI research database was encrypted and de-identified to protect their privacy.

### Study cohort and outcomes

This case–control study extracted data from the NHI program in Taiwan from January 2004 to December 2017 using newly diagnosed type 2 DM codes based on the International Classification of Diseases, ninth revision, Clinical Modification (ICD-9-CM) and ICD, tenth revision, CM (ICD-10-CM). The newly diagnosed type 2 DM was defined as the first time that a type 2 DM code was available in the outpatient or inpatient claim records between January 2004 and December 2017. The list of ICD-9 and ICD-10 codes that used to defined the inclusion of T2DM patients, study events, and co-morbidities were presented in Additional file 1: Table S1.

**Fig. 1:**
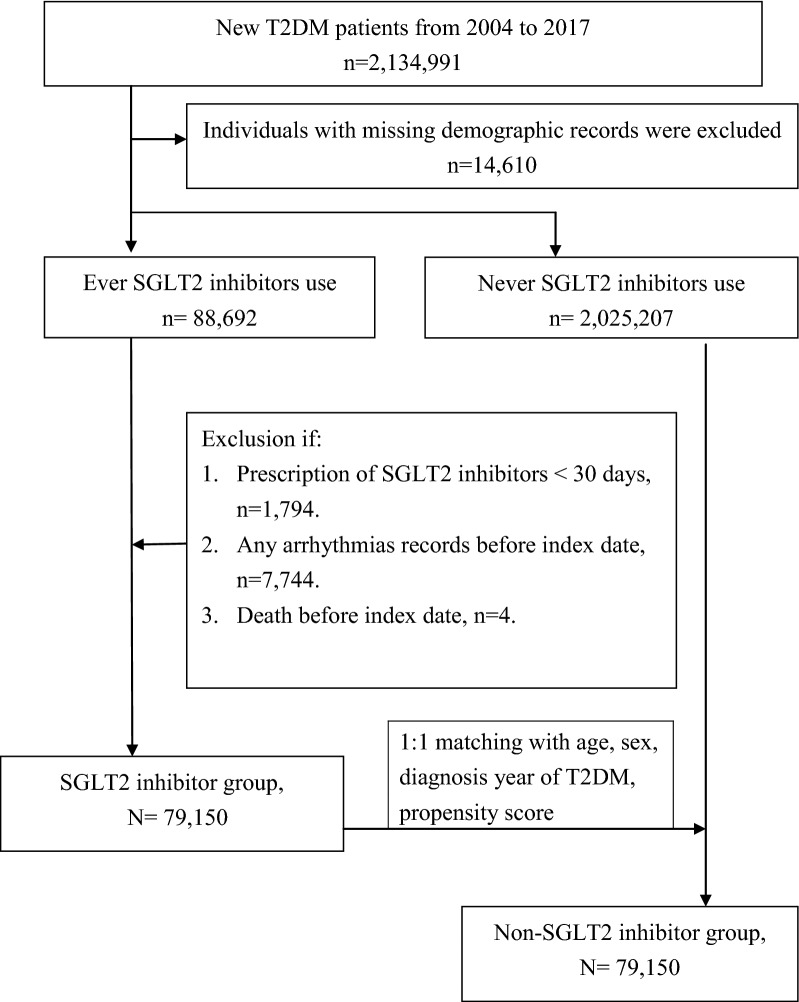
Flowchart of study population

A flowchart for the enrollment of the study cohort is summarized in Fig. 1. Study subjects were divided into two groups: those taking SGLT2 inhibitors (the SGLT2 inhibitors group, *n* = 95,174) and those not taking SGLT2 inhibitors (the non-SGLT2 inhibitor group, *n *= 2,025,207). Among the SGLT2 inhibitor group, 9542 patients were excluded because of a diagnosis of any arrhythmias (*n* = 7744) before the index date, use of SGLT2 inhibitor for less than 1 month (*n* = 1794), and death (*n* = 4). A total of 79,150 patients were selected for this study.

Both the SGLT2 inhibitor and non-SGLT2 inhibitor groups were stratified in a 1:1 ratio. The study group comprised 79,150 participants with SGLT2 inhibitors as antihyperglycemic drugs, and the control group included 79,150 age-, sex-, DM duration-, drug index date-, and propensity score-matched randomly selected participants without SGLT2 inhibitors.

The first claim date of the SGLT2 inhibitors was defined as the drug index date in the SGLT2 inhibitor group, with the same day of the matched non-SGLT2 inhibitor group. Two types of SGLT2 inhibitors (empagliflozin and dapagliflozin) were launched on May 1, 2016 and their till to end of study (Dec. 31, 2017). Median (Q1–Q3, Maximum) of follow up time was 9 (4–14, 21), in SGLT2 inhibitors group was 9 (5, 14), in non-SGLT2 inhibitors group was 9 (4, 14). In NHIRD, the records of prescriptions were uploaded when the medications were filled. We followed the SGLT2 inhibitors users since their first prescription of SGLT2 inhibitors, until the occurrence of study event or end of study. The intention to treat analysis was performed in this study, we analyzed according to the group they were originally assigned, regardless of their adherence or duration on usage of SGLT2 inhibitor. The study outcome was defined by the all-cause mortality and new-onset arrhythmias (NOA), including atrial fibrillation, atrial flutter, atrial premature complexes, paroxysmal supraventricular tachycardia, ventricular tachycardia, ventricular fibrillation, and ventricular premature complexes, based on the ICD-9-CM and ICD-10-CM codes, in either an outpatient or inpatient department at least once from May 1, 2016 to December 31, 2017.

### Statistical analysis

Data are presented as valid percentages and mean values with a standard deviation. The standardized difference was applied to determine the difference in baseline characteristics between the two study groups. The propensity score method was used to compare the effect between the two study groups on study outcomes. Inverse probability of treatment weighting of propensity scores was used to balance covariates across the two study groups [17]. The balance of potential confounders at baseline (index date) between the two study groups was evaluated using absolute standardized difference, rather than statistical testing, because balance is a property of the sample and not of an underlying population. A value of the absolute standardized difference ≤ 0.1 suggested a negligible difference in potential confounders between the two study groups (Table 1). The Cox proportional hazard regression model was used to compare the risk of developing study events between the SGLT2 inhibitor group and the non-SGLT2 inhibitor group. Adjusted hazard ratios (HRs) and 95% confidence intervals (CIs) were calculated, adjusting for important risk factors for developing study events, including age, sex, medication use, and comorbidities. The risk of study outcomes overtime for the SGLT2 inhibitor group compared with the non-SGLT2 inhibitor group was determined using survival analysis with the Kaplan–Meier method. Finally, the Cox regression model was used as a sensitivity analysis by age ≥ 60 or < 60 years. All effects were analyzed using an intention-to-treat approach. Statistical significance was defined at a P-value of < 0.05.

**Table 1:** Baseline characteristics among study cohorts before and after propensity score matching

	Before PSM			After PSM		
	Non-SGLT2i	SGLT2i	ASD	Non-SGLT2i	SGLT2i	ASD
	N = 319,848	N = 79,962		N = 79,150	N = 79,150	
Year of T2DM diagnosis			0.000			0.082
2004	29,528 (9.23%)	7382 (9.23%)		7643 (9.66%)	7311 (9.24%)	
2005	25,620 (8.01%)	6405 (8.01%)		6515 (8.23%)	6347 (8.02%)	
2006	25,508 (7.98%)	6377 (7.98%)		6586 (8.32%)	6322 (7.99%)	
2007	26,372 (8.25%)	6593 (8.25%)		6646 (8.4%)	6518 (8.23%)	
2008	26,040 (8.14%)	6510 (8.14%)		6594 (8.33%)	6454 (8.15%)	
2009	26,920 (8.42%)	6730 (8.42%)		6653 (8.41%)	6664 (8.42%)	
2010	24,684 (7.72%)	6171 (7.72%)		5948 (7.51%)	6102 (7.71%)	
2011	23,324 (7.29%)	5831 (7.29%)		5577 (7.05%)	5761 (7.28%)	
2012	22,500 (7.03%)	5625 (7.03%)		5312 (6.71%)	5562 (7.03%)	
2013	21,528 (6.73%)	5382 (6.73%)		5123 (6.47%)	5317 (6.72%)	
2014	19,976 (6.25%)	4994 (6.25%)		4574 (5.78%)	4941 (6.24%)	
2015	18,872 (5.90%)	4718 (5.90%)		4491 (5.67%)	4661 (5.89%)	
2016	17,108 (5.35%)	4277 (5.35%)		4356 (5.50%)	4244 (5.36%)	
2017	11,868 (3.71%)	2967 (3.71%)		3132 (3.96%)	2946 (3.72%)	
Sex			0.000			0.014
Male	179,252 (56.04%)	44,813 (56.04%)		44,751 (56.54%)	44,360 (56.05%)	
Female	140,596 (43.96%)	35,149 (43.96%)		34,399 (43.46%)	34,790 (43.95%)	
Age			0.000			0.039
< 50	99,056 (30.97%)	24,764 (30.97%)		24,179 (30.55%)	24,471 (30.92%)	
50–60	105,012 (32.83%)	26,253 (32.83%)		26,379 (33.33%)	26,037 (32.90%)	
60–70	87,708 (27.42%)	21,927 (27.42%)		21,897 (27.67%)	21,705 (27.42%)	
≥ 70	28,072 (8.78%)	7018 (8.78%)		6695 (8.46%)	6937 (8.76%)	
Comorbidities						
Hypertension	155,971 (48.76%)	47,428 (59.31%)	0.213	47,636 (60.18%)	46,839 (59.18%)	0.021
Hyperlipidemia	163,107 (51.00%)	55,788 (69.77%)	0.391	57,253 (72.33%)	55,107 (69.62%)	0.060
Cirrhosis	7638 (2.39%)	2152 (2.69%)	0.019	2070 (2.62%)	2117 (2.67%)	0.004
COPD	8870 (2.77%)	2009 (2.51%)	0.016	1932 (2.44%)	1983 (2.51%)	0.004
Sleep apnea	1516 (0.47%)	584 (0.73%)	0.033	512 (0.65%)	561 (0.71%)	0.008
Concurrent medication						
NSIADs (exclude aspirin)	68,643 (21.46%)	16,491 (20.62%)	0.021	16,423 (20.75%)	16,349 (20.66%)	0.002
Systemic corticosteroids	17,933 (5.61%)	4258 (5.33%)	0.012	4192 (5.3%)	4203 (5.31%)	0.001
PPIs	20,853 (6.52%)	5043 (6.31%)	0.009	4860 (6.14%)	4984 (6.3%)	0.006
H2 receptor inhibitors	37,135 (11.61%)	9502 (11.88%)	0.008	9262 (11.7%)	9391 (11.86%)	0.005
Aspirins	49,502 (15.48%)	18,635 (23.30%)	0.199	17,980 (22.72%)	18,278 (23.09%)	0.009
Biguanides	141,971 (44.39%)	54,094 (67.65%)	0.482	55,453 (70.06%)	53,339 (67.39%)	0.058
Sulfonylureas	95,541 (29.87%)	37,970 (47.49%)	0.368	38,563 (48.72%)	37,359 (47.2%)	0.030
Alpha glucosidase inhibitors	27,832 (8.7%)	16,654 (20.83%)	0.347	15,144 (19.13%)	16,149 (20.4%)	0.032
Thiazolidinediones	26,241 (8.2%)	14,809 (18.52%)	0.307	13,905 (17.57%)	14,393 (18.18%)	0.016
DPP4is	54,820 (17.14%)	34,119 (42.67%)	0.581	32,709 (41.33%)	33,358 (42.15%)	0.017
Insulin	23,666 (7.40%)	14,210 (17.77%)	0.317	13,001 (16.43%)	13,716 (17.33%)	0.024
Alpha-blockers	9202 (2.88%)	2590 (3.24%)	0.021	2522 (3.19%)	2564 (3.24%)	0.003
Beta-blockers	64,898 (20.29%)	21,416 (26.78%)	0.153	20,747 (26.21%)	21,057 (26.6%)	0.009
CCBs	76,458 (23.90%)	19,026 (23.79%)	0.003	19,097 (24.13%)	18,848 (23.81%)	0.007
ACEIs	17,276 (5.40%)	5415 (6.77%)	0.057	5403 (6.83%)	5349 (6.76%)	0.003
ARBs	119,902 (37.49%)	42,612 (53.29%)	0.321	42,504 (53.7%)	41,975 (53.03%)	0.013
Statins	148,755 (46.51%)	57,867 (72.37%)	0.546	58,549 (73.97%)	57,092 (72.13%)	0.042

The comorbidities were defined by disease diagnosis (ICD-9 or ICD-10) that listed in Additional file 1: Table S1

*PSM* propensity score matching, *SGLT2i* Sodium–glucose cotransporter 2 inhibitors, *ASD* absolute standardized difference, *COPD* chronic obstructive pulmonary disease, *PVD* peripheral vascular disease, *PCI* percutaneous coronary intervention, *NSIAD* non-steroidal anti-inflammatory drug, *PPI* proton pump inhibitor, *DPP-4i* dipeptidyl peptidase 4 inhibitor, *CCB* calcium channel blocker, *ACEI* angiotension-converting enzyme inhibitor, *ARB* angiotensin receptor blocker

## Results

In total, 79,150 pairs were included after age, sex, DM duration, drug index date, and propensity score matching. The baseline characteristics of all patients between the SGLT2 inhibitor group and the non-SGLT2 inhibitor group are presented in Table 1. The absolute standardized differences between the two groups in all variables were < 0.1 (10%), and the differences between matched pairs were statistically negligible.

**Fig. 2:**
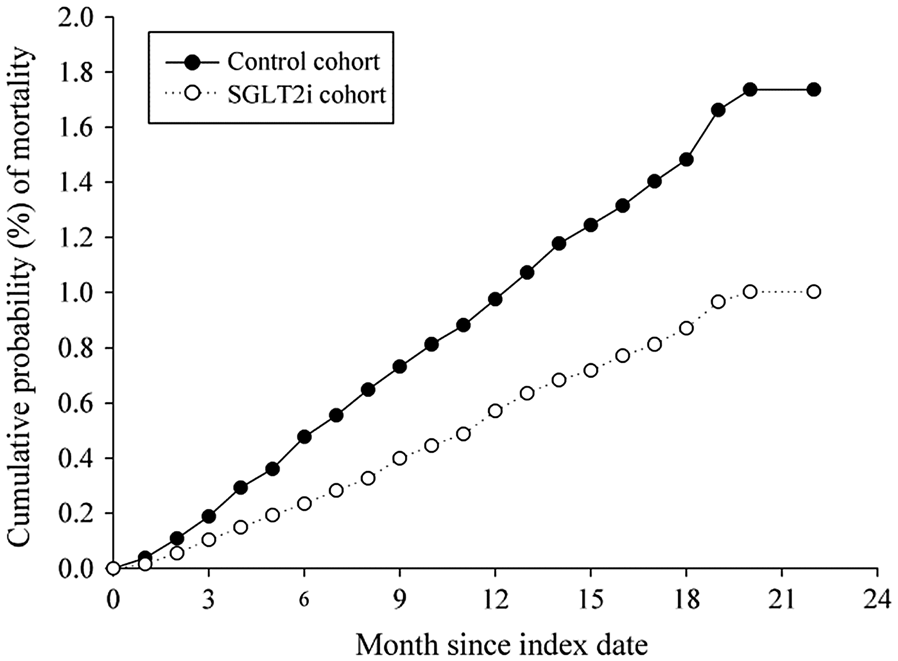
Cumulative risk curve of all-cause mortality for the study cohorts treated with SGLT2 inhibitors vs. non-SGLT2 inhibitors user

**Fig. 3:**
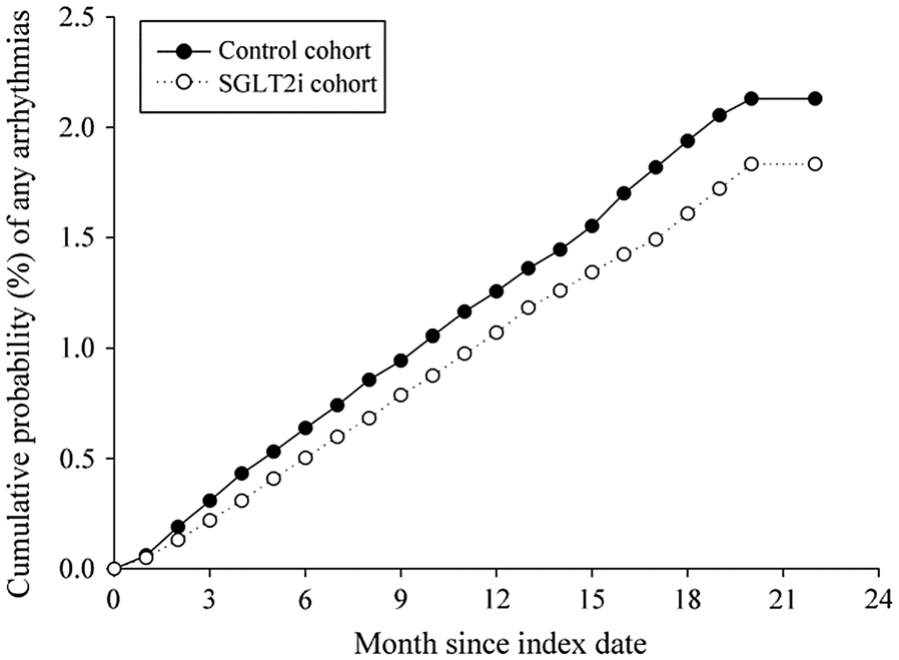
Cumulative risk curve of new-onset any arrhythmias for the study cohorts treated with SGLT2 inhibitors vs. non-SGLT2 inhibitors users

In total, 1046 all-cause death events were observed among the matched patients during the follow-up period. The SGLT2 inhibitor group was associated with a lower risk of all-cause mortality [adjusted hazard ratio (aHR): 0.547; 95% confidential interval (CI) 0.482–0.621; *P* = 0.0001]. Total events of NOA were 1579, including atrial fibrillation (*n* = 271), supraventricular arrhythmias (*n* = 121), and ventricular arrhythmias (*n* = 91). The aHRs of NOA were 0.830 (95% CI 0.751–0.916; *P* = 0.002) in the SGLT2 inhibitor group. The events and incidence rate of all-cause death and NOA are shown in Table 2. There is a clear separation of event curves for all-cause mortality and NOA between these two groups, as shown in Figs. 2 and 3.

**Table 2:** Incidence of study events in population between SGLT2 inhibitor group and non- SGLT2 inhibitor group

	Non-SGLT2i		SGLT2i		cHR	aHR
	Pm	Events	Pm	Events		
All-cause death	733,896	666	742,390	380	0.564 (0.497–0.640)	0.547 (0.482–0.621)
NOA	728,545	858	737,979	721	0.832 (0.754–0.919)	0.830 (0.751–0.916)
Atrial fibrillation	733,017	146	741,713	125	0.849 (0.668–1.077)	0.841 (0.662–1.068)
Supraventricular arrhythmias	733,479	67	742,054	54	0.799 (0.558–1.143)	0.815 (0.570–1.167)
Ventricular arrhythmias	733,651	50	742,207	41	0.813 (0.538–1.229)	0.797 (0.525–1.208)

aHR, controlled for age, sex, comorbidities, and concurrent medications

Supraventricular arrhythmias include atrial premature complexes and paroxysmal supraventricular tachycardia; ventricular arrhythmias include ventricular tachycardia, ventricular fibrillation and ventricular premature complexes

*SGLT2i* sodium–glucose co-transporter 2 inhibitor, *Pm* Person-months, *cHR* crude hazard ratio, *aHR* adjusted hazard ratio, *NOA* new-onset arrhythmias

We performed several sensitivity analyses to investigate the effect of SGLT2 inhibitor on all-cause death and NOA. Exclusion of all NOA events for participants of an all-cause death event within 60 years old provided similar results (147 vs. 238 events, HR 0.47, 95% CI 0.35 to 0.63; Table 3). Similar findings were seen when participants aged ≥ 60 years did not experience NOA at any time during the study (233 vs. 361 events; HR 0.57, 95% CI 0.45 to 0.72; Table 3). Similarly, a consistent treatment effect of NOA was seen when participants age within 60 years did not experience an all-cause death event (369 vs. 452 events; HR 0.81, 95% CI 0.67 to 0.99) or at any time during the study (352 vs. 406 events; HR 0.83, 95% CI 0.68 to 1.01; Table 3).

**Table 3:** Sensitivity analyses

Outcome	Non-SGLT2i			SGLT2i			Hazard Ratio	P value
	%	20 M KM (%)	Events/10,000 Ptms	%	20 KM (%)	Events/10,000 Ptms	(95% CI)	
Age < 60 year and All-cause death	305 (0.60%)	0.60	6.42	147 (0.29%)	0.29	3.05	0.47 (0.35–0.63)	< 0.001
Age ≥ 60 year and All-cause death	361 (1.26%)	1.26	13.94	233 (0.81%)	0.81	8.93	0.57 (0.45–0.72)	< 0.001
Age < 60 year and NOA	452 (0.89%)	0.87	9.58	369 (0.73%)	0.72	7.70	0.81 (0.67–0.99)	0.042
Age ≥ 60 year and NOA	406 (1.42%)	1.40	15.82	352 (1.23%)	1.21	13.60	0.83 (0.68–1.01)	0.061

*NOA* new-onset arrhythmias, *10,000 Ptms* per 10,000 patient months, *SGLT2i* sodium–glucose cotransporter 2 inhibitors

## Discussion

In this population-based cohort study, we observed that patients taking SGLT2 inhibitors had a significantly lower risk of all-cause mortality compared with non-SGLT2 inhibitor users. The risk of NOA among the diabetes population taking SGLT2 inhibitors was decreased by 17% compared with non-SGLT2 inhibitor users.

### Cardiovascular protective benefit

SGLT2, which is significantly increased in patients with type 2 DM, is located at the S1 and S2 segments of the proximal tubule epithelium and responsible for around 90% of filtered glucose reabsorption in the kidney. SGLT2 inhibitors inhibit the reabsorption of filtered glucose, thus increasing glycosuria and also natriuresis [18–20]. The EMPA-REG OUTCOME study and CANVAS program have shown the cardiovascular protection benefit of lowering the risk of cardiovascular death or hHF in type 2 diabetic patients with high risk or established cardiovascular diseases [8, 9]. Furthermore, the Dapagliflozin on the Incidence of Cardiovascular Events (DECLARE) and Dapagliflozin And Prevention of Adverse-outcomes in Heart Failure (DAPA-HF) trials demonstrated the cardiovascular protective benefit of lowering the risk of cardiovascular death or hHF in patients with type 2 DM with a lower rate of major adverse cardiac events (MACE), and a first worsening heart failure event [21, 22]. In our study, SGLT2 inhibitor users reduced all-cause mortality by 46% compared with non-SGLT2 inhibitor users, which is consistent with the large randomized control trial and observational study [8, 23, 24]. In real-world practice, the multinational CVD-REAL study has shown the lower rate of hHF and death with SGLT2 inhibitor treatment compared with other antihyperglycemic drugs [23]. Simultaneously, a nationwide retrospective observational study that estimated the effect of SGLT2 inhibitors on hHF among diabetes patients suggests that SGLT2 inhibitors reduced hHF compared with dipeptidyl peptidase-4 inhibitors. The benefit of reduced hHF was noted as earlier as 30 days after initiating the SGLT2 inhibitors among patients with established cardiovascular diseases [25]. The lower risk of hHF occurred during the early phase of the SGLT2 inhibitor initiation, suggesting that patients with heart failure are predisposed to develop arrhythmias. Otherwise, arrhythmias can exacerbate the heart failure symptoms and increase the risk of hHF by decreasing the effective cardiac output [26, 27].

### Protective effect of arrhythmia

In the present cohort study, we observed that patients taking SGLT2 inhibitors had a potential protective effect of NOA. Till now, little data are available regarding therapeutic strategies to improve prognosis in these patients. The recent EMPA-REG OUTCOME study showed that administration of the SGLT2 inhibitor, empagliflozin, significantly suppressed cardiovascular death, including sudden cardiac death [8]. This study reported pleiotropic effects, including hypotension and bodyweight reduction, as a result of SGLT2 inhibitor administration. The physiological mechanisms involved in SGLT2 inhibitor administration and cardiac arrhythmia, however, remain unknown. Currently, an observational study by Zelniker et al. reported that the risk of atrial fibrillation or atrial flutter was as low as 19% in patients using empagliflozin [28]. They suggested a possible antiarrhythmic effect of SGLT2 inhibitors, including structural, electromechanical, and mechanical myocardial remodeling and an imbalance in the sympathetic-parasympathetic tone.

To our knowledge, our study is the first real-world data to describe the risk of arrhythmias in DM patients taking SGLT2 inhibitors. Our study suggests that patients with diabetes taking SGLT2 inhibitors can reduce the 17% risk of arrhythmias development compared with non-SGLT2 inhibitor users, which may be the possible cause of decreasing the risk of hHF observed in current trials.

### Strengthens and limitations

The strengthens of our study included a population-based nature, large sample size, and de-identified data. Our study also has are several limitations. First, the shortest follow-up period in our participants is 20 months. However, the results of all-cause mortality and arrhythmias still showed statistical significance. Second, the laboratory data such as hemoglobin A1c levels, blood sugar levels, renal function, liver function, and electrocardiogram data were not available from these secondary data. This is an important limitation. However, because the data we used were population-based data, we assumed that there were no differences among the two groups. Further randomized clinical trial is needed to confirm our result. Third, we ascertained that the exposure to SGLT2 inhibitors in the cohort is real and supported by the claims data, which include medication prescription. However, treatment adherence was not available from these secondary data. Fourth, the present study was based on a retrospective review of prescription records, which quite naturally were not adequate source of information for specifically looking at cardiac arrhythmias. Hence, it is quite likely that cardiac arrhythmias were underreported at the time of inclusion. However, our results are consistent with those of previous validation studies [29–32]. Fifth, we could not exclude the possibility of time lag bias in this analysis. However, the index date in the control group is the same calendar day as that of the matched SGLT2 inhibitor group. It has about the same amount of time into follow-up after the type 2 DM diagnosis. Therefore, we assumed that there is a low possibility of time lag bias in this analysis.

## Conclusion

Patients with type 2 DM taking SGLT2 inhibitors as antihyperglycemic drugs are associated with a lower risk of all-cause mortality and arrhythmias compared with those without SGLT2 inhibitors prescription in real-world practice.

## Supplementary information

**Additional file 1: Table S1.** The lists of ICD-9 and ICD-10 codes in inclusion criteria, study events, and co-morbidities**. Table S2.** Subgroup analyses for sex. **Table S3.** Subgroup analyses for age. **Table S4.** Subgroup analyses for DM duration. **Table S5.** Subgroup analyses for comorbidity. **Table S6.** Subgroup analyses for current medication.

## Data Availability

All the re-identified data are available upon reasonable request (cgp8009@yahoo.com.tw).

## Abbreviations

SGLT: Sodium–glucose co-transporter
hHF: Hospitalization for heart failure
T2DM: Type 2 diabetes mellitus
ICD-9-CM: International Classification of Diseases, ninth revision; Clinical Modification
ICD-10-CM: International Classification of Diseases, tenth revision, Clinical Modification Clinical Modification
AF: Atrial fibrillation
RAS: Renin–angiotensin system
