# Supplementary material for: The association between SGLT2 inhibitors and new-onset arrhythmias: a nationwide population-based longitudinal cohort study
Source: Cardiovasc Diabetol. 2020 Jun 5;19:73. doi: 10.1186/s12933-020-01048-x (PMC7275510; doi:10.1186/s12933-020-01048-x)
Supplement: Supplementary file 1 — Additional file 1: Table S1. The lists of ICD-9 and ICD-10 codes in inclusion criteria, study events, and co-morbidities. Table S2. Subgroup analyses for sex. Table S3. Subgroup analyses for age. Table S4. Subgroup analyses for DM duration. Table S5. Subgroup analyses for comorbidity. Table S6. Subgroup analyses for current medication. [file 12933_2020_1048_MOESM1_ESM.docx]

Additional Tables

Table S1. The lists of ICD-9 and ICD-10 codes in inclusion criteria, study events, and co-morbidities

| Item | ICD-9 | ICD-10 |
| --- | --- | --- |
| **Inclusion criteria** |  |  |
| Type 2 DM | 250.x (excluding 250.x1, 250.x3) | E11 |
| **Outcome** |  |  |
| Any arrhythmias | 427.0x, 427.1x, 427.2x, 427.3x, 427.4x, 427.6x, 427.69, 427.9x | I47-I49 |
| Atrial fibrillation or atrial flutter | 427.3x | I48 |
| Supraventricular arrhythmias | 427.0x, 427.61 | I47.1, I49.1, I49.2 |
| Ventricular arrhythmias | 427.1x, 427.4x | I47.0, I47.2, I49.3, I49.0 |
| **Comorbidities** |  |  |
| Hypertension | 401–405 | I10, I11, I12, I13, I15 |
| Hyperlipidemia | 272.x (excluding 272.5x, 272.8) | E78.0- E78.5 |
| Cirrhosis | 571.5 | K74 |
| COPD | 490–492, 496 | J40-J44 |
| Sleep apnea | 780.51, 780.57, 327.23, 780.53 | G47.3 |

Table S2 Subgroup analyses for sex

|  | Non-SGLT2 | | SGLT2 | |  |
| --- | --- | --- | --- | --- | --- |
|  | Event (%) | Rate, per 10000 person-months | Event (%) | Rate, per 10000 person-months | aHR |
| **All cause of death** |  |  |  |  |  |
| Male | 463(1.035%) | 11.31(10.33-12.39) | 260(0.586%) | 6.32(5.59-7.13) | 0.543(0.466-0.633) |
| Female | 203(0.590%) | 6.25(5.45-7.18) | 120(0.345%) | 3.63(3.03-4.34) | 0.572(0.455-0.720) |
| p for interaction |  |  |  |  | 0.8155 |
| **Arrhythmia** |  |  |  |  |  |
| Male | 444(0.992%) | 10.92(9.95-11.98) | 361(0.814%) | 8.82(7.95-9.77) | 0.794(0.691-0.914) |
| Female | 414(1.204%) | 12.86(11.68-14.16) | 360(1.035%) | 10.96(9.88-12.15) | 0.870(0.754-1.002) |
| p for interaction |  |  |  |  | 0.3468 |

Table S3 Subgroup analyses for age

|  | Non-SGLT2 | | SGLT2 | |  |
| --- | --- | --- | --- | --- | --- |
|  | Event (%) | Rate, per 10000 person-months | Event (%) | Rate, per 10000 person-months | aHR |
| **All cause of death** |  |  |  |  |  |
| <50 | 134(0.554%) | 5.88(4.97-6.97) | 52(0.212%) | 2.22(1.69-2.91) | 0.394(0.284-0.547) |
| 50-60 | 171(0.648%) | 6.92(5.96-8.04) | 95(0.365%) | 3.85(3.15-4.70) | 0.560(0.434-0.724) |
| 60-70 | 207(0.945%) | 10.34(9.03-11.85) | 116(0.534%) | 5.82(4.85-6.98) | 0.544(0.432-0.686) |
| >=70 | 154(2.300%) | 26.17(22.35-30.65) | 117(1.687%) | 19.03(15.88-22.82) | 0.651(0.505-0.839) |
| p for interaction |  |  |  |  | 0.0767 |
| **Arrhythmia** |  |  |  |  |  |
| <50 | 182(0.753%) | 8.03(6.94-9.29) | 154(0.629%) | 6.60(5.63-7.72) | 0.829(0.668-1.028) |
| 50-60 | 270(1.024%) | 11.01(9.77-12.40) | 215(0.826%) | 8.75(7.66-10.00) | 0.801(0.668-0.959) |
| 60-70 | 284(1.297%) | 14.31(12.74-16.08) | 224(1.032%) | 11.31(9.92-12.89) | 0.792(0.663-0.945) |
| >=70 | 122(1.822%) | 20.98(17.57-25.06) | 128(1.845%) | 21.06(17.71-25.05) | 0.966(0.749-1.246) |
| p for interaction |  |  |  |  | 0.4695 |

Table S4 Subgroup analyses for DM duration

|  | Non-SGLT2 | | SGLT2 | |  |
| --- | --- | --- | --- | --- | --- |
|  | Event (%) | Rate, per 10000 person-months | Event (%) | Rate, per 10000 person-months | aHR |
| **All cause of death** |  |  |  |  |  |
| <2 years | 52(0.674%) | 7.17(5.46-9.41) | 37(0.459%) | 4.88(3.54-6.74) | 0.625(0.404-0.967) |
| 2-5 years | 88(0.617%) | 6.56(5.32-8.08) | 65(0.429%) | 4.56(3.58-5.82) | 0.624(0.446-0.872) |
| >=5 years | 526(0.938%) | 9.98(9.16-10.87) | 278(0.499%) | 5.30(4.72-5.97) | 0.524(0.452-0.607) |
| p for interaction |  |  |  |  | 0.4519 |
| **Arrhythmia** |  |  |  |  |  |
| <2 years | 76(0.991%) | 10.55(8.42-13.20) | 61(0.760%) | 8.09(6.29-10.39) | 0.707(0.502-0.995) |
| 2-5 years | 124(0.874%) | 9.30(7.80-11.09) | 118(0.782%) | 8.32(6.95-9.97) | 0.854(0.663-1.101) |
| >=5 years | 658(1.182%) | 12.58(11.65-13.58) | 542(0.978%) | 10.41(9.57-11.32) | 0.835(0.745-0.936) |
| p for interaction |  |  |  |  | 0.7322 |

Table S5 Subgroup analyses for comorbidity

|  | Non-SGLT2 | | SGLT2 | |  |
| --- | --- | --- | --- | --- | --- |
|  | Event (%) | Rate, per 10000 person-months | Event (%) | Rate, per 10000 person-months | aHR |
| **All cause of death** |  |  |  |  |  |
| Hypertension=0 | 213(0.676%) | 7.36(6.43-8.42) | 115(0.356%) | 3.81(3.17-4.57) | 0.492(0.391-0.619) |
| Hypertension=1 | 453(0.951%) | 10.19(9.30-11.18) | 265(0.566%) | 6.02(5.34-6.79) | 0.581(0.499-0.677) |
| p for interaction |  |  |  |  | 0.2133 |
| Hyperlipidemia=0 | 295(1.347%) | 14.73(13.14-16.51) | 159(0.661%) | 7.13(6.10-8.32) | 0.489(0.403-0.594) |
| Hyperlipidemia =1 | 371(0.648%) | 6.95(6.28-7.70) | 221(0.401%) | 4.26(3.73-4.86) | 0.619(0.524-0.732) |
| p for interaction |  |  |  |  | 0.0883 |
| Liver cirrhosis =0 | 603(0.782%) | 8.57(7.92-9.29) | 345(0.448%) | 4.86(4.37-5.40) | 0.549(0.481-0.628) |
| Liver cirrhosis =1 | 63(3.043%) | 20.64(16.12-26.42) | 35(1.653%) | 10.82(7.77-15.07) | 0.523(0.332-0.824) |
| p for interaction |  |  |  |  | 0.6893 |
| COPD =0 | 606(0.785%) | 8.47(7.82-9.17) | 348(0.451%) | 4.81(4.33-5.34) | 0.556(0.487-0.636) |
| COPD =1 | 60(3.106%) | 32.96(25.59-42.45) | 32(1.614%) | 16.81(11.88-23.77) | 0.511(0.322-0.809) |
| p for interaction |  |  |  |  | 0.6122 |
| **Arrhythmia** |  |  |  |  |  |
| Hypertension=0 | 287(0.911%) | 9.98(8.89-11.20) | 229(0.709%) | 7.62(6.69-8.67) | 0.748(0.627-0.891) |
| Hypertension=1 | 571(1.199%) | 12.95(11.93-14.06) | 492(1.050%) | 11.25(10.30-12.29) | 0.874(0.774-0.986) |
| p for interaction |  |  |  |  | 0.1509 |
| Hyperlipidemia=0 | 236(1.078%) | 11.87(10.45-13.49) | 235(0.977%) | 10.60(9.33-12.04) | 0.887(0.739-1.064) |
| Hyperlipidemia =1 | 622(1.086%) | 11.74(10.85-12.70) | 486(0.882%) | 9.41(8.61-10.29) | 0.811(0.72-0.914) |
| p for interaction |  |  |  |  | 0.4764 |
| Liver cirrhosis =0 | 824(1.069%) | 11.80(11.02-12.63) | 690(0.896%) | 9.78(9.07-10.53) | 0.828(0.748-0.917) |
| Liver cirrhosis =1 | 34(1.643%) | 11.25(8.04-15.75) | 31(1.464%) | 9.66(6.79-13.74) | 0.908(0.535-1.543) |
| p for interaction |  |  |  |  | 0.9367 |
| COPD =0 | 818(1.059%) | 11.51(10.75-12.33) | 688(0.892%) | 9.57(8.88-10.31) | 0.832(0.752-0.922) |
| COPD =1 | 40(2.070%) | 22.34(16.39-30.45) | 33(1.664%) | 17.52(12.45-24.64) | 0.717(0.430-1.196) |
| p for interaction |  |  |  |  | 0.7437 |

Table S6 Subgroup analyses for current medication

|  | Non-SGLT2 | | SGLT2 | |  |
| --- | --- | --- | --- | --- | --- |
|  | Event (%) | Rate, per 10000 person-months | Event (%) | Rate, per 10000 person-months | aHR |
| **All cause of death** |  |  |  |  |  |
| NSIADs=0 | 506(0.807%) | 8.73(8.00-9.53) | 292(0.465%) | 4.96(4.42-5.57) | 0.547(0.473-0.634) |
| NSIADs =1 | 160(0.974%) | 10.37(8.88-12.10) | 88(0.538%) | 5.72(4.64-7.05) | 0.522(0.401-0.679) |
| p for interaction |  |  |  |  | 0.7615 |
| steroids=0 | 545(0.727%) | 7.84(7.20-8.52) | 315(0.420%) | 4.48(4.01-5.00) | 0.560(0.487-0.644) |
| steroids=1 | 121(2.886%) | 31.56(26.41-37.72) | 65(1.547%) | 16.59(13.01-21.16) | 0.502(0.369-0.681) |
| p for interaction |  |  |  |  | 0.6578 |
| PPI =0 | 562(0.756%) | 8.14(7.50-8.84) | 331(0.446%) | 4.75(4.26-5.29) | 0.569(0.496-0.652) |
| PPI =1 | 104(2.140%) | 23.88(19.70-28.94) | 49(0.983%) | 10.82(8.17-14.31) | 0.463(0.325-0.658) |
| p for interaction |  |  |  |  | 0.1615 |
| Aspirin =0 | 451(0.737%) | 7.97(7.27-8.74) | 248(0.407%) | 4.36(3.85-4.94) | 0.528(0.451-0.618) |
| Aspirin =1 | 215(1.196%) | 12.78(11.18-14.60) | 132(0.722%) | 7.61(6.42-9.02) | 0.597(0.479-0.743) |
| p for interaction |  |  |  |  | 0.4439 |
| Biguanides =0 | 245(1.034%) | 11.20(9.88-12.69) | 139(0.539%) | 5.51(4.67-6.51) | 0.545(0.441-0.675) |
| Biguanides =1 | 421(0.759%) | 8.17(7.43-8.99) | 241(0.452%) | 4.92(4.33-5.58) | 0.558(0.475-0.654) |
| p for interaction |  |  |  |  | 0.7501 |
| Sulfonylureas =0 | 345(0.850%) | 9.40(8.46-10.45) | 178(0.426%) | 4.54(3.92-5.26) | 0.492(0.409-0.591) |
| Sulfonylureas =1 | 321(0.832%) | 8.75(7.84-9.76) | 202(0.541%) | 5.77(5.03-6.62) | 0.609(0.509-0.727) |
| p for interaction |  |  |  |  | 0.0736 |
| Thiazolidinediones =0 | 541(0.829%) | 8.98(8.25-9.77) | 302(0.466%) | 5.02(4.49-5.62) | 0.545(0.472-0.628) |
| Thiazolidinediones =1 | 125(0.899%) | 9.52(7.99-11.34) | 78(0.542%) | 5.54(4.44-6.91) | 0.57(0.426-0.762) |
| p for interaction |  |  |  |  | 0.9674 |
| DPP4=0 | 344(0.741%) | 8.04(7.24-8.94) | 219(0.478%) | 5.25(4.60-5.99) | 0.579(0.487-0.688) |
| DPP4=1 | 322(0.984%) | 10.51(9.43-11.73) | 161(0.483%) | 4.95(4.25-5.78) | 0.486(0.400-0.590) |
| p for interaction |  |  |  |  | 0.2144 |
| Insullin =0 | 450(0.680%) | 7.36(6.71-8.08) | 273(0.417%) | 4.51(4.01-5.08) | 0.565(0.486-0.658) |
| Insullin =1 | 216(1.661%) | 17.59(15.39-20.10) | 107(0.780%) | 7.79(6.44-9.41) | 0.485(0.384-0.613) |
| p for interaction |  |  |  |  | 0.2569 |
| Beta- blockers =0 | 425(0.728%) | 7.87(7.15-8.65) | 246(0.423%) | 4.52(3.99-5.12) | 0.556(0.474-0.652) |
| Beta- blockers =1 | 241(1.162%) | 12.44(10.97-14.12) | 134(0.636%) | 6.78(5.72-8.03) | 0.537(0.433-0.666) |
| p for interaction |  |  |  |  | 0.9369 |
| CCBs =0 | 441(0.734%) | 7.95(7.24-8.73) | 249(0.413%) | 4.38(3.87-4.96) | 0.533(0.456-0.623) |
| CCBs =1 | 225(1.178%) | 12.55(11.01-14.30) | 131(0.695%) | 7.54(6.35-8.95) | 0.596(0.478-0.743) |
| p for interaction |  |  |  |  | 0.454 |
| ACEI /ARB=0 | 247(0.761%) | 8.09(7.15-9.17) | 147(0.440%) | 4.68(3.98-5.51) | 0.548(0.446-0.674) |
| ACEI /ARB =1 | 419(0.919%) | 9.77(8.88-10.75) | 233(0.511%) | 5.44(4.78-6.18) | 0.557(0.474-0.655) |
| p for interaction |  |  |  |  | 0.9197 |
| Statin =0 | 262(1.272%) | 13.62(12.07-15.38) | 154(0.698%) | 7.57(6.47-8.87) | 0.545(0.446-0.666) |
| Statin =1 | 404(0.690%) | 7.46(6.77-8.22) | 226(0.396%) | 4.19(3.68-4.78) | 0.566(0.480-0.667) |
| p for interaction |  |  |  |  | 0.8027 |
| **Arrhythmia** |  |  |  |  |  |
| NSIADs=0 | 612(0.976%) | 10.63(9.82-11.51) | 506(0.806%) | 8.64(7.92-9.43) | 0.807(0.717-0.908) |
| NSIADs =1 | 246(1.498%) | 16.10(14.21-18.24) | 215(1.315%) | 14.09(12.32-16.10) | 0.872(0.724-1.049) |
| p for interaction |  |  |  |  | 0.5026 |
| steroids=0 | 776(1.035%) | 11.23(10.47-12.05) | 668(0.891%) | 9.55(8.86-10.31) | 0.850(0.766-0.943) |
| steroids=1 | 82(1.956%) | 21.69(17.47-26.93) | 53(1.261%) | 13.65(10.43-17.87) | 0.629(0.444-0.892) |
| p for interaction |  |  |  |  | 0.1291 |
| PPI =0 | 773(1.041%) | 11.28(10.51-12.10) | 639(0.862%) | 9.22(8.53-9.96) | 0.817(0.735-0.907) |
| PPI =1 | 85(1.749%) | 19.76(15.98-24.45) | 82(1.645%) | 18.33(14.76-22.76) | 0.929(0.681-1.267) |
| p for interaction |  |  |  |  | 0.4066 |
| Aspirin =0 | 559(0.914%) | 9.94(9.15-10.80) | 461(0.757%) | 8.14(7.43-8.92) | 0.818(0.722-0.926) |
| Aspirin =1 | 299(1.663%) | 17.97(16.05-20.13) | 260(1.422%) | 15.13(13.40-17.08) | 0.848(0.716-1.003) |
| p for interaction |  |  |  |  | 0.7221 |
| Biguanides =0 | 274(1.156%) | 12.62(11.21-14.21) | 253(0.980%) | 10.09(8.92-11.41) | 0.867(0.730-1.030) |
| Biguanides =1 | 584(1.053%) | 11.42(10.53-12.38) | 468(0.877%) | 9.61(8.77-10.52) | 0.818(0.724-0.925) |
| p for interaction |  |  |  |  | 0.7625 |
| Sulfonylureas =0 | 450(1.109%) | 12.35(11.26-13.55) | 359(0.859%) | 9.20(8.30-10.21) | 0.768(0.667-0.883) |
| Sulfonylureas =1 | 408(1.058%) | 11.20(10.17-12.34) | 362(0.969%) | 10.40(9.39-11.53) | 0.908(0.787-1.048) |
| p for interaction |  |  |  |  | 0.0706 |
| Thiazolidinediones =0 | 722(1.107%) | 12.07(11.22-12.98) | 583(0.900%) | 9.75(8.99-10.57) | 0.808(0.724-0.902) |
| Thiazolidinediones =1 | 136(0.978%) | 10.42(8.81-12.33) | 138(0.959%) | 9.86(8.34-11.65) | 0.922(0.725-1.173) |
| p for interaction |  |  |  |  | 0.2959 |
| DPP4=0 | 506(1.090%) | 11.92(10.93-13.01) | 386(0.843%) | 9.30(8.42-10.27) | 0.775(0.678-0.885) |
| DPP4=1 | 352(1.076%) | 11.58(10.43-12.85) | 335(1.004%) | 10.38(9.32-11.55) | 0.918(0.788-1.069) |
| p for interaction |  |  |  |  | 0.0964 |
| Insullin =0 | 712(1.076%) | 11.74(10.91-12.63) | 585(0.894%) | 9.73(8.97-10.55) | 0.818(0.733-0.913) |
| Insullin =1 | 146(1.123%) | 11.97(10.18-14.08) | 136(0.992%) | 9.96(8.42-11.78) | 0.918(0.725-1.163) |
| p for interaction |  |  |  |  | 0.4481 |
| Beta- blockers =0 | 486(0.832%) | 9.04(8.27-9.89) | 425(0.732%) | 7.84(7.13-8.62) | 0.872(0.765-0.994) |
| Beta- blockers =1 | 372(1.793%) | 19.45(17.57-21.53) | 296(1.406%) | 15.11(13.48-16.93) | 0.786(0.673-0.917) |
| p for interaction |  |  |  |  | 0.376 |
| CCBs =0 | 568(0.946%) | 10.31(9.50-11.19) | 497(0.824%) | 8.79(8.05-9.59) | 0.852(0.755-0.962) |
| CCBs =1 | 290(1.519%) | 16.33(14.56-18.32) | 224(1.188%) | 13.00(11.40-14.82) | 0.789(0.661-0.942) |
| p for interaction |  |  |  |  | 0.4874 |
| ACEI /ARB=0 | 311(0.964%) | 10.26(9.18-11.46) | 252(0.759%) | 8.07(7.13-9.13) | 0.796(0.674-0.941) |
| ACEI /ARB =1 | 547(1.209%) | 12.86(11.83-13.98) | 469(1.035%) | 11.01(10.06-12.06) | 0.854(0.754-0.967) |
| p for interaction |  |  |  |  | 0.3772 |
| Statin =0 | 216(1.048%) | 11.31(9.90-12.93) | 207(0.938%) | 10.24(8.94-11.74) | 0.893(0.736-1.083) |
| Statin =1 | 642(1.097%) | 11.94(11.05-12.90) | 514(0.900%) | 9.59(8.80-10.46) | 0.810(0.721-0.910) |
| p for interaction |  |  |  |  | 0.4948 |
